# State-of-the-art mobile head CT scanner delivers nearly the same image quality as a conventional stationary CT scanner

**DOI:** 10.1038/s41598-024-56089-z

**Published:** 2024-03-16

**Authors:** Lukas Goertz, Yosef Al-Sewaidi, Mahmoud Habib, David Zopfs, Benjamin Reichardt, Alexander Ranft, Christoph Kabbasch

**Affiliations:** 1 Department of Radiology and Neuroradiology, Faculty of Medicine and University Hospital, University of Cologne, Kerpener Straße 62, 50937 Cologne, Germany; 2 Department of Interventional Radiology and Neuroradiology, Klinikum Hochsauerland, Arnsberg, Germany

**Keywords:** Computed tomography, ICU, Intracranial haemorrhage, Mobile CT, Trauma, Brain injuries, Brain imaging, Radiography, Three-dimensional imaging, Tomography

## Abstract

The use of mobile head CT scanners in the neurointensive care unit (NICU) saves time for patients and NICU staff and can reduce transport-related mishaps, but the reduced image quality of previous mobile scanners has prevented their widespread clinical use. This study compares the image quality of SOMATOM On.Site (Siemens Healthineers, Erlangen, Germany), a state-of-the-art mobile head CT scanner, and a conventional 64-slice stationary CT scanner. The study included 40 patients who underwent head scans with both mobile and stationary scanners. Gray and white matter signal and noise were measured at predefined locations on axial slices, and signal-to-noise ratios (SNRs) and contrast-to-noise ratios (CNRs) were calculated. Artifacts below the cranial calvaria and in the posterior fossa were also measured. In addition, image quality was subjectively assessed by two radiologists in terms of corticomedullary differentiation, subcalvarial space, skull artifacts, and image noise. Quantitative measurements showed significantly higher image quality of the stationary CT scanner in terms of noise, SNR and CNR of gray and white matter. Artifacts measured in the posterior fossa were higher with the mobile CT scanner, but subcalvarial artifacts were comparable. Subjective image quality was rated similarly by two radiologists for both scanners in all domains except image noise, which was better for stationary CT scans. The image quality of the SOMATOM On.Site for brain scans is inferior to that of the conventional stationary scanner, but appears to be adequate for daily use in a clinical setting based on subjective ratings.

## Introduction

Neurointensive care units (NICUs) are highly specialized facilities designed to provide intensive care for individuals with critical neurological conditions, including ischemic stroke, traumatic brain injury, and spontaneous intracranial hemorrhage, and postoperative surveillance^1,2^. In this context, computed tomography (CT) serves as an important diagnostic tool, enabling rapid and accurate identification of life-threatening conditions such as increased intracranial pressure caused by cerebral edema, cerebral hemorrhage, or hydrocephalus.

Standard practice is to transfer patients from the NICU to the radiology department for a CT scan using a stationary scanner. However, this process poses logistical and staffing challenges, as it is essential to ensure continuous supply and monitoring of various life-supporting elements such as pumps and ventilation during transport^3^. Unfortunately, transport-related mishaps occur can occur in up to 30% of cases, such as inadvertent removal of ventilator access, disconnection of monitoring equipment, or interruption of medication delivery^4^.

On the contrary, the introduction of a mobile CT scanner in the NICU reduces the risks associated with patient repositioning, speeds up imaging and ensures that the full range of critical care therapies remains readily available in the event of complications^5^.

While earlier mobile CT scanners offered compromised image quality due to their compact design^6^, advances in CT technology may have improved the quality of these scanners.

The present study retrospectively compares the image quality of a state-of-the-art mobile head CT scanner, the SOMATOM On.Site (Siemens Healthineers, Erlangen, Germany), with that of a conventional stationary CT scanner used at the same institution. The comparison was made on a per-patient basis using both subjective assessments and objective measurements.

## Results

### Patient characteristics

During the study period, approximately 350 head CT scans were performed on NICU patients, including 107 (30.6%) mobile CT scans. Thereof, a total of 40 patients (age: 69.4 ± 17.2 years, 55% female) met the inclusion criteria and were enrolled into this study. Among these patients, the most common reasons for admission to the NICU were post-trauma monitoring in 10 patients (25%) and postoperative monitoring in 10 patients (25%). The most common reason for mobile CT imaging was postoperative neurosurgical monitoring in 16 patients (40%), followed by intracranial hemorrhage monitoring in 13 (33%) and neurological deterioration in 8 (20%). Detailed patient characteristics are presented in Table 1.

**Table 1:** Baseline patient characteristics.

Parameter	Value (n = 40)
Patient age (years), mean ± SD	69.4 ± 17.2
Female sex, N (%)	22 (55%)
Reasons for NICU admission	
Postoperative surveillance, N (%)	10 (25%)
Spontaneous ICH, N (%)	7 (18%)
Posttraumatic surveillance, N (%)	10 (25%)
Ischemic stroke, N (%)	2 (5%)
Hydrocephalus, N (%)	3 (8%)
Miscellaneous, N (%)	8 (20%)
Reasons for mobile head CT	
Postoperative neurosurgical control, N (%)	16 (40%)
Control of EVD location, N (%)	1 (3%)
Control of intracranial bleedings, N (%)	13 (33%)
Follow-up of ischemic stroke, N (%)	2 (5%)
Neurological worsening/deterioration, N (%)	8 (20%)

*N* number, *SD* standard deviation, *NICU* neurointensive care unit, *ICH* intracerebral hemorrhage, *CT* computed tomography, *EVD* external ventricular drain.

### Imaging quality

**Figure 1:**
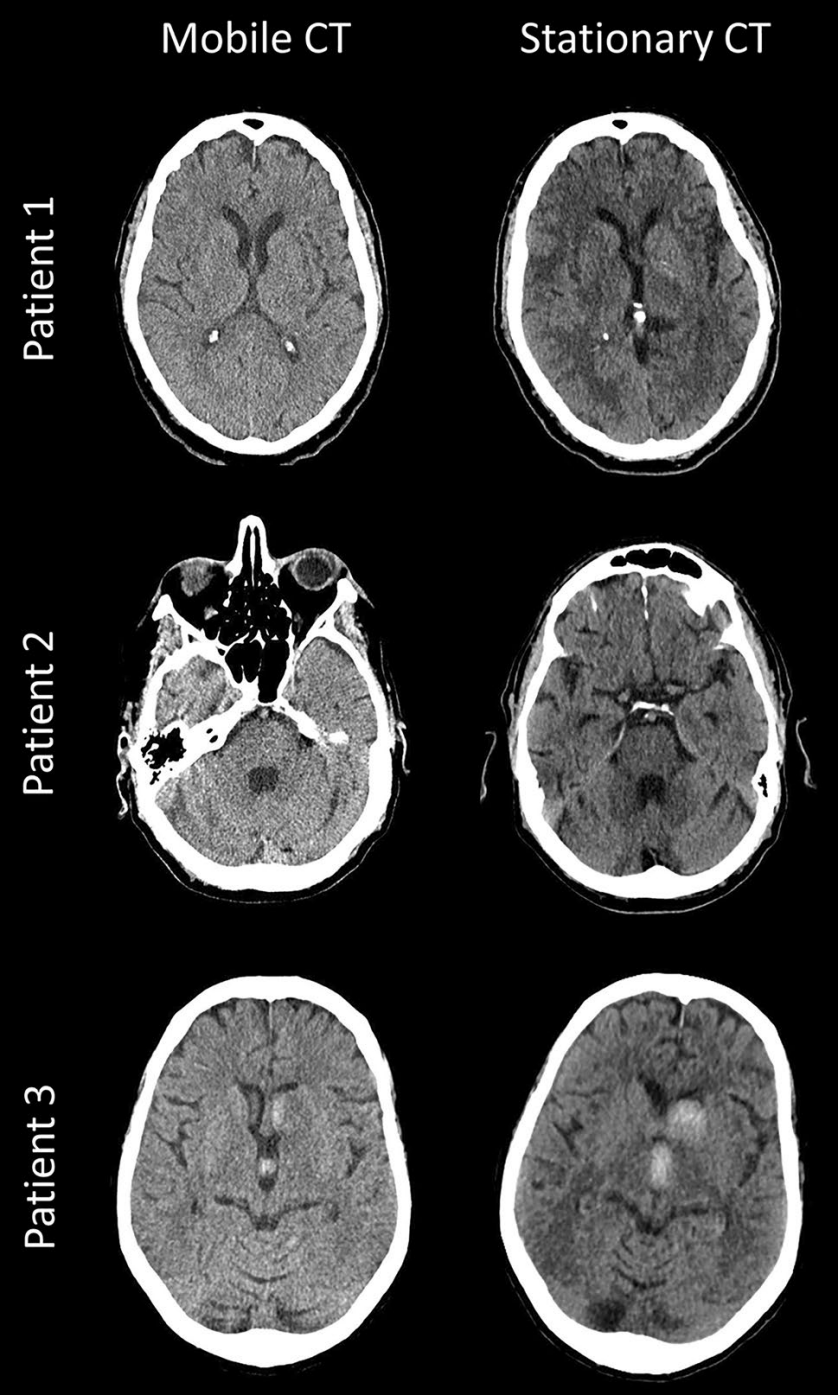
Intra-individual comparison between mobile and stationary head CT scans of illustrative cases, (**A**) of the cerebrum (patient 1), (**B**) of the posterior fossa (patient 2), and (**C**) of the cerebrum with basal ganglia hemorrhage and ventricular hemorrhage (patient 3).

The mean dose length product was 939 ± 132 mGy*cm for mobile CT and 862 ± 181 mGy*cm for stationary CT (p = 0.03). Illustrative CT scans showing the image quality of mobile and CT scanners are shown in Fig. 1.

**Figure 2:**
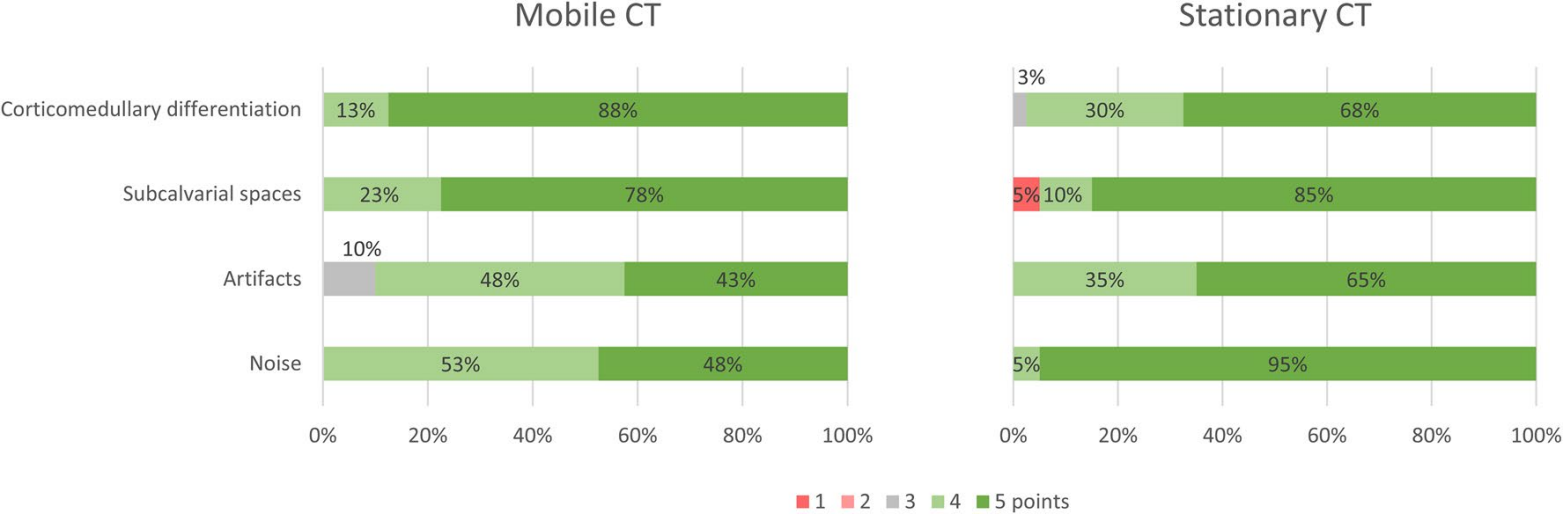
Subjective image quality rating on a 5-point Likert scale (1 = poor to 5 = excellent) for corticomedullary differentiation, subcalvarial spaces, beam hardening artifacts, and image noise.

Subjective assessment of image quality (Table 2) showed comparable scores for corticomedullary differentiation, subcalvarial space, and beam hardening artifacts. Only for image noise, the mobile head CT scan was rated worse than the stationary CT scan (p = 0.01). Overall, a score of 4 or 5 was given in the majority of cases, as shown in Fig. 2. Interrater agreement ranged from slight (0.20) to moderate (0.57), indicating diverging opinions (Table 2).

**Table 2:** Subjective assessment of image quality, presented as the mean of readers 1 and 2 (range: 1–5).

	Mobile CT		Stationary CT		P-value
	Median (IQR)	Kendall's τ	Median (IQR)	Kendall's τ	
Corticomedullary differentiation	5 (5–5)	0.37	5 (4–5)	0.44	0.26
Subcalvarial space	5 (5–5)	0.35	5 (5–5)	0.57	0.50
Beam hardening artifacts	4 (4–5)	0.41	5 (4–5)	0.48	0.10
Image noise	4 (4–5)	0.20	5 (5–5)	0.54	0.01

*IQR* interquartile range.

Kendall's τ represents the interrater agreement between the two readers.

Quantitative measurements of image quality (Table 3) showed that the imaging noise of both grey and white matter was consistently significantly lower in stationary CT scans than in mobile CT scans for all measured regions (p < 0.01 for each). Overall, the signal-to-noise ratio (SNR) of gray matter was higher in stationary CT scans, but statistical significance was not reached for each region, as detailed in Table 3. In contrast the SNR of white matter was higher in mobile CT scans (p < 0.01). Gray/white matter contrast-to-noise ratio (CNR) was better in stationary than in mobile CT scans, also without reaching statistical significant difference for all regions. Posterior fossa beam hardening artifacts (PFAI) were lower in the stationary CT scans (p = 0.01), while subcalvarial artifacts (SAI) were comparable between the two scanner types (p = 0.63). Intraclass correlation was lower for the mobile CT (median: 0.44) than for the stationary CT (median: 0.82, p < 0.01).

**Table 3:** Quantitative evaluation of image quality and comparison between mobile and stationary CT scanners.

Quantitative measurement of imaging quality	Mobile CT		Stationary CT		P-value
	Mean (SD)	ICC	Mean (SD)	ICC	
Gray matter noise					
Overall	4.6 ± 0.8	0.47	3.7 ± 0.6	0.81	< 0.01
Frontal	4.2 ± 0.7	0.23	3.6 ± 0.6	0.90	< 0.01
Parietal	4.3 ± 0.6	0.30	3.3 ± 0.5	0.74	< 0.01
Basal ganglia	5.2 ± 1.0	0.75	4.2 ± 0.6	0.83	< 0.01
Gray matter SNR					
Overall	9.8 ± 2.1	0.31	10.7 ± 2.4	0.68	0.01
Frontal	10.8 ± 2.3	0.21	11.0 ± 2.7	0.72	0.75
Parietal	10.7 ± 2.6	0.13	12.8 ± 2.9	0.56	< 0.01
Basal ganglia	7.8 ± 1.4	0.68	8.3 ± 1.4	0.78	0.08
White matter noise					
Overall	4.2 ± 0.7	0.51	3.5 ± 0.6	0.86	< 0.01
Frontal	3.9 ± 0.7	0.55	3.4 ± 0.5	0.85	< 0.01
Parietal	4.1 ± 0.7	0.42	3.4 ± 0.6	0.87	< 0.01
Basal ganglia	4.7 ± 0.8	0.75	3.8 ± 0.6	0.88	< 0.01
White matter SNR					
Overall	7.9 ± 1.5	0.47	7.4 ± 1.8	0.72	< 0.01
Frontal	8.6 ± 1.7	0.42	7.5 ± 1.6	0.78	< 0.01
Parietal	8.1 ± 1.3	0.32	7.6 ± 2.1	0.66	0.19
Basal ganglia	7.1 ± 1.4	0.68	7.2 ± 1.6	0.78	0.75
Gray matter-white matter CNR					
Overall	3.2 ± 0.8	0.35	4.4 ± 1.0	0.84	< 0.01
Frontal	3.6 ± 0.9	0.44	4.4 ± 0.9	0.81	< 0.01
Parietal	3.7 ± 0.9	0.36	5.7 ± 1.1	0.88	< 0.01
Basal ganglia	2.5 ± 0.7	0.44	3.1 ± 0.9	0.84	< 0.01
SAI	4.6 ± 1.2	0.83	4.8 ± 1.8	0.90	0.63
PFAI	6.4 ± 0.9	0.97	5.2 ± 0.8 s	0.92	< 0.01

*SD* standard deviation, *SNR* signal-to-noise ratio, *CNR* contrast-to-noise ratio, *SAI* subcalvarial artifact index, *PFAI* posterior fossa artifact index, *ICC* intraclass correlation coefficient.

## Discussion

This study investigated the image quality of head CT scans obtained using a mobile scanner and a conventional stationary scanner. The evaluation included established objective image criteria such as SNR of gray and white matter and CNR in various brain regions, as well as a subjective assessment of image quality. The results showed that the conventional stationary CT scanner outperformed the mobile scanner in terms of noise, gray matter SNR, and GM-WM CNR. However, the mobile scanner was superior for SNR of white matter. The stationary scanner had fewer posterior fossa artifacts compared to the mobile CT scanner, while subcalvarial artifacts were comparable between the two scanners.

### Imaging quality

Good CNR is important because differentiation between gray and white matter on CT is difficult due to minimal differences in HU attenuation. However, differentiation is important to evaluate a variety of pathologies such as cerebral edema, territorial infarcts, and tumors.

The quantitative results indicated slightly better imaging quality of the stationary CT scanner over the SOMATOM On.Site. Similarly, using a different measurement method, Andersson previously reported slightly lower image quality from the same mobile CT scanner compared to the current generation of stationary CT scanners^7^.

In the current study, the specific image quality measures for mobile CT were: GM noise 4.6, WM noise 4.2, GM SNR 9.8, WM SNR 7.9, GWM CNR 3.2, and PFAI 6.4. Comparing these results to previous publications with similar methodological assessment of image quality, Pomerantz et al. reported the following values for a 64-row stationary CT scanner (Discovery CT750 HD, GE Healthcare; Chicago, IL, USA): GM noise 4.6, WM noise 4.2, GM SNR 8.0, WM SNR 6.2, GWM CNR 1.5, and PFAI 11.7^8^. Notably, while the image quality of the mobile scanner was inferior to that of the stationary scanner in the present study, the SNR, CNR, and PFAI of the Somatom On.Site were intermediate to better than that of the stationary CT scanner in the study by Pomerantz et al. published in 2013. However, current state-of-the-art stationary CT scanners achieve even better imaging quality than that of the stationary CT scanner in the present study (GM noise 3.7, WM noise 3.5, GM SNR 10.7, WM SNR 7.4, GWM CNR 4.4, and PFAI 5.2). In 2017, Neuhaus et al. reported the following values for a dual-energy spectral CT on polyenergetic images (IQon Spectral CT, Philips, Best, the Netherlands): GM noise 2.8, WM noise 2.7, GM SNR 12.4, WM SNR 10.5, CNR 2.7, SAI 4.2, and PFAI 6^9^. The use of monoenergetic reconstructions could further improve these values. Using a photon-counting CT scanner (Naeotom Alpha, Siemens Healthineers, Erlangen, Germany), Michael et al. reported even better values in 2022: GM noise: 2.2, WM noise: 2.1, GM SNR: 20.1, WM SNR: 16.8, GM-WM-CNR: 2.9, SAI: 3.9, and PFAI: 9.7^10^.

These results clearly demonstrate how CT scanner technology has improved over the past decade. In this respect, the concept of mobile head CT scanners is not entirely new, but the initial investment cost and reduced image quality of earlier scanners have prevented their widespread use in clinical practice^6^. The Philips Tomoscan M, one of the first portable CT scanners, had image quality that was inferior to that of the stationary CT scanners of the time, in particular increased image noise^11,12^. It was also relatively large, difficult to position in the ICU, and had a reputation for technical unreliability. Abdullah et al. compared the image quality of the CereTom CT scanner (introduced in 2004) with that of a stationary CT scanner from the same year and reported significantly lower image quality of the mobile CT, particularly in terms of radiation artifacts, gray-white matter differentiation, and delineation of intracranial lesions^6^.

While the quantitative measurements in this study showed the superiority of the stationary over the mobile CT scanner, the readers rated the subjective image quality of both scanners as equivocal in all categories except image noise. These results suggest that the small differences in quantitative image quality assessment may not necessarily interfere with radiologic evaluation. However, it must be considered that some observer bias is likely, as blinded analysis is not possible when comparing mobile and stationary CT scanners. This may have led to an overestimation of mobile CT images and a devaluation of stationary CT images. In this context, the interrater agreement was only slight to moderate, indicating diverging opinions. Nevertheless, the results suggest that the image quality of the SOMATOM On.Site appears to be sufficient for routine clinical use, especially in the acute NICU setting where timely diagnosis is critical, especially as it offers certain advantages over stationary CT scans for use in the NICU.

### Advantages and drawbacks of mobile CT scanners

The major advantage of mobile CT is that it brings imaging directly to the patient's bedside for immediate use and can expedite the diagnosis of critical brain conditions. Conversely, the standard approach requires patient preparation, transport to the radiology department and multiple repositioning, which increases intracranial pressure^13^. In critically ill patients, delays of more than an hour can occur in diagnosis and vital therapeutic interventions such as EVD application, evacuation of intracranial hemorrhage, or hemicraniectomy. There is substantial evidence that patient morbidity increases when treatment of elevated intracranial pressure is delayed^14–17^.

Bringing imaging to the patient saves time for patients and also reduces NICU staff workload. Studies by Masaryk et al. showed a 30-min reduction in overall NICU staff workload with CT scanners, potentially reducing costs and burden^18^. Similarly, Gunnarsson et al. observed a significant reduction in individual staff workload, resulting in a 145-min reduction in nursing time for high-risk patients and a 64-min reduction for low-risk patients^3^. However, the use of mobile CT scanners increases the workload of radiology staff due to time-consuming tasks such as transport and data processing^19^.

Mobile CT scanners minimize patient transport mishaps by scanning only the patient's head. In comparison, Smith et al. reported up to 30% mishaps during repositioning and transport with stationary CT scanners^4^. Gunnarsson et al. found 4.3% complications in high-risk patients with mobile CT scanners compared to 25% with stationary scanners^3^. In this context, patient positioning and transport can lead to blood pressure changes, desaturation and increased intracranial pressure^3^. Prolonged immobilisation and flat lying can lead to higher ICP, which over time can lead to secondary brain damage and worse outcomes^14,16^.

Mobile CT scanners also have limitations. They are mainly suitable for imaging the head and brain, not for detailed imaging of other parts of the body. Comparison of acquired studies with previous studies is difficult because they are not readily available on the scanner. Although computed tomography angiography and perfusion imaging are possible with the SOMATOM On.Site, these studies require specialised equipment and have not been established in our department.

There is also the problem of inadequate radiation shielding. As a result, other patients in the room have to be moved or protected with portable lead walls^19^. In addition, the average radiation exposure to patients was about 9% higher for mobile CT than for stationary CT. However, this slight increase seems acceptable, provided that it speeds up diagnosis and possible treatment.

While mobile head CT saves time for NICU staff, it requires more time from the radiologic technologist. A mini-poll of 5 radiologic technologists in the authors' department showed that the time required for a mobile CT scan, from organising the scan to archiving it in the PACS and returning to the workstation, was approximately 45 to 60 min, compared with 20 to 30 min for a stationary scan. The radiologic technologist also needs special training on the mobile CT scanner. At the authors' institution, mobile CT scans are performed by the regular radiologic technologist staff. Therefore, if the radiology department is very busy or if trained radiologic technologists are not available, a mobile CT scan cannot be performed. Mobile CT scans are also not performed after normal working hours or at weekends. Given these limitations, approximately 30% of NICU head scans were performed with the mobile scanner at the authors' institution during the study period.

Considering these aspects, the image quality of the SOMATOM On.Site seems to be of adequate diagnostic quality for everyday head imaging on the NICU, such as the diagnosis or control of intracranial haemorrhage or rebleeding. It is clear that the imaging quality is inferior to that of state-of-the-art stationary CT scanners, but it brings the advantages of faster diagnosis, reduction of transport mishaps and relief of NICU staff. Therefore, the choice of CT scanner in clinical practice depends not only on imaging quality, but also on individual requirements and the purpose of the study.

### Limitations

The study has several limitations. The study group was assembled retrospectively and is relatively small and heterogeneous. Lack of prior experience with mobile CT in our group may have affected image quality. The stationary CT scanner used in this study is not the latest CT scanner technology, as there have been several technical advances since its introduction, including spectral CT and photon-counting CT. To partially mitigate this limitation, we reported image quality values from recent studies with comparable image quality assessment methods. Finally, because the study design does not allow for blinded analysis, both objective and especially subjective measurements may be subject to observation bias, resulting in an overestimation of mobile CT and an underestimation of stationary CT. Despite this limitation, we believe that the SOMATOM On.Site may provide sufficient image quality to justify its use in modern NICUs in daily practice.

## Conclusions

The results of this comparative analysis indicate a slightly better image quality of conventional stationary CT scanners compared to a state-of-the-art mobile CT scanner. However, this small advantage in image quality does not necessarily translate into radiological image interpretation. Because mobile CT scanners can save NICU staff time and reduce patient transport and positioning mishaps, they may be beneficial for NICU patients when rapid imaging is needed.

## Methods

This is a retrospective, comparative study of patients who underwent mobile head CT imaging in a multidisciplinary NICU at a tertiary care academic teaching hospital between May 2021 and March 2023. Consecutive patients were screened for specific inclusion criteria: (1) successful technical performance of mobile head CT covering the neurocranium, (2) availability of institutional stationary CT, (3) absence of midline shift, (4) at least one hemisphere and the posterior fossa without apparent pathology such as territory infarction, intracranial hemorrhage, cerebral edema, tumor, or pneumocephalus; and (5) and presence of implants on both scans, if applicable. All data were collected retrospectively and anonymized. The study received institutional review board approval and informed consent in accordance with institutional policy. The study was conducted in accordance with the STROBE guidelines in compliance with the national legislation and the Code of Ethical Principles for Medical Research Involving Human Subjects of the World Medical Association (Declaration of Helsinki).

### Siemens SOMATOM On.site mobile CT scanner

**Figure 3:**
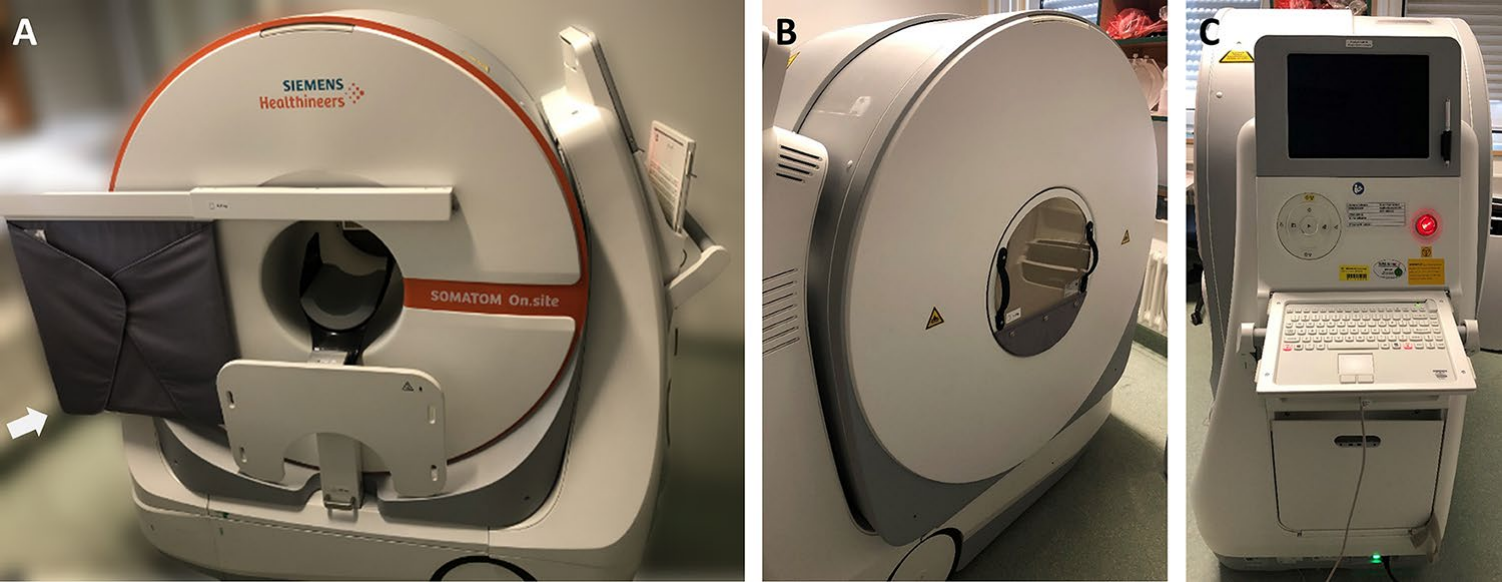
Images of the Siemens SOMATOM On.site taken at the storage location in the NICU with frontal (**A**), oblique (**B**) and lateral (**C**) views. The CT scanner weighs 890 kg but can be moved and operated by one person. It is equipped with a keyboard and touch screen for operating the unit and viewing reconstructed images of the scan at the bedside (**C**). A lead apron is fixed to the scanner (**A**, block arrow), which can be moved for radiation protection of the adjacent patient.

The SOMATOM On.site weighs 890 kg (plus 6.4 kg for the lead apron and 3.5 kg for the lead glass). It has dimensions of 160 × 74 × 155 cm (W × D × H) and a field of view of 26 cm. The standard scan length is 170 mm (maximum 200 mm) and covers the head to the 5th cervical vertebra. The scanner has a 35 cm gantry opening and a 32-row detector with a spatial resolution of 0.75 mm. Tube voltages range from 80 to 120 kV, and the study used a tube voltage of 120 kV and a tube current of 38 mAs, resulting in a CTDI of 44.09 mGy for all cases. The scanner has a rotation time of one second and a tilt factor of 0.55. It has a telescopic gantry with an inner part (X-ray tube and detector) that moves during scanning at a feed rate of 14.2 mm/s (max 32 mm/s). In the current study, scans were performed with the following parameters: (a) slice thickness of 1 mm with triplanar reconstructions with a slice thickness of 3 mm; (b) pitch of 0.55; (c) collimation of 32 × 0.75 mm; (d) increment of 1 mm (reconstructed increment of 3 mm); (e) rotation time of 1 s; (f) CT dose index volume of 44.09 mGy; and (g) 38 mAs tube current–time product and 120 kV tube voltage. Raw data were then processed using either a soft tissue or bone kernel. Post-processing was performed using the SAFIRE or iMAR iterative reconstruction algorithms. Photos of the SOMATOM On.site are shown in Fig. 3.

### Stationary CT scanner

All stationary CT scans were performed with the Aquilion Lightning SP (Canon Medical Solutions, Neuss, Germany). Head scans were performed with the following parameters: (a) slice thickness of 1 mm with triplanar reconstructions with a slice thickness of 3 mm; (b) pitch of 0.6; (c) collimation of 80 × 0.5 mm; (d) increment of 1 mm (reconstructed increment of 3 mm); (e) rotation time of 1.5 s; (f) CT dose index volume of 44.09 mGy (avg. 43.27); (g) tube current–time product of 117–300 mAs (average 239.45 mAs) and h) tube voltage of 120–130 kV (average 120.25 kV). Raw data were then processed using a standard kernel for both soft tissue and bone. Post-processing was performed using the iterative reconstruction algorithm AIDR 3D or SEMAR for metal artifact suppression.

### Data collection and assessment of imaging quality

The following data were retrospectively collected: patient age, sex, reasons for NICU stay and CT imaging performance, and calculated radiation exposure (dose-length product).

For subjective analysis, two radiologists (Y.A. and M.H.) with 4 and 5 years of experience in interpreting head CT scans subjectively rated image quality using a 5-point Likert scale. They evaluated corticomedullary differentiation (1 = difficult, uncertain diagnosis; 2 = poor, limited diagnosis; 3 = fair, diagnostic; 4 = good, diagnostic; 5 = excellent, fully diagnostic), subcalvarial space evaluation (ranging from 1 = not evaluable/uncertain diagnosis to 5 = unrestricted evaluable/fully diagnostic), beam hardening artifacts caused by the skull (ranging from 1 = massive to 5 = none), and image noise (ranging from 1 = excessive to 5 = sharp images without noise).

**Figure 4:**
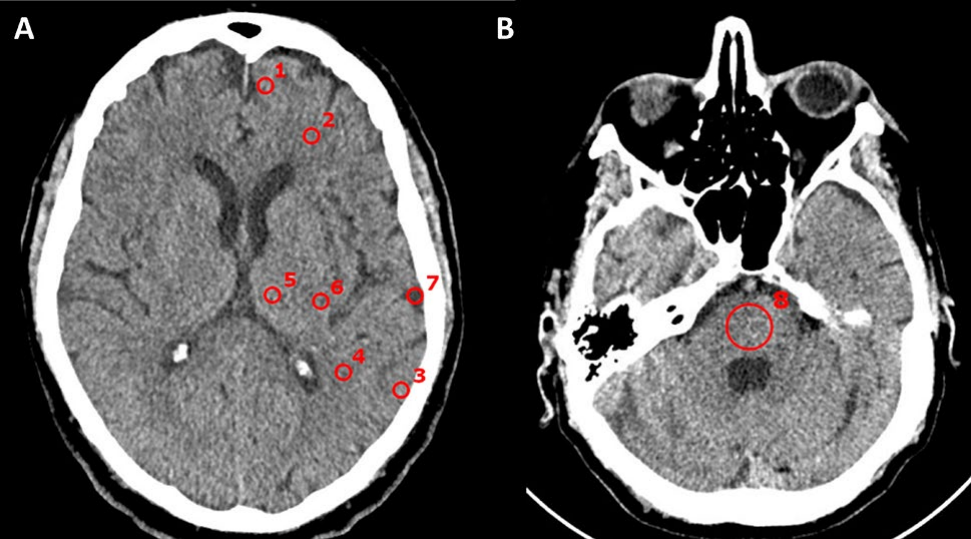
Placement of regions of interest: (**A**) in the frontal (1) and adjacent frontal white matter (2), parietal cortex (3) and adjacent parietal white matter (4), thalamus (5) and posterior internal capsule (6), and gray matter just below the cranial calvaria (7); (**B**) at the level between the petrous bones, in the central part of the pons (8).

For quantitative measurements of cerebral image quality, eight regions of interest (ROIs) were positioned on an axial plane and included the cortex of the frontal (a) and parietal (b) lobes, the thalamus (c), the juxtacortical white matter of the frontal (d) and parietal (e) lobes, the posterior limb of the internal capsule (f), a region near the calvarium (g), and the medulla oblongata in a plane between the petrous parts of the temporal bones (h). The measurements are visualized in Fig. 4.

The position and dimensions of the ROIs remained consistent between mobile and stationary CT scans. ROIs in the gray and white matter, thalamus, and internal capsule were uniformly set at 25 mm^2^, with adjustments made to avoid including tissues of varying attenuation. The ROI in the medulla oblongata was 200 mm^2^.

Image quality parameters were calculated as previously described^10,20^. The standard deviation (SD) in Hounsfield units within each ROI was considered as an indicator of image noise. The SDs in measurements between the petrous parts of the temporal bones were termed posterior fossa artifact index (PFAI). The SD of a ROI placed near the calvarium indicates skull-induced beam hardening artifacts and is referred to as the subcalvarial artifact index (SAI). The signal-to-noise ratio (SNR) was calculated by dividing the average Hounsfield units by the SD within a single ROI, while the contrast-to-noise ratio (CNR) was determined between adjacent ROIs by calculating the difference between the average Hounsfield units for the two regions and dividing by the square root of the sum of their SDs.

### Statistical analysis

Qualitative parameters are presented as numbers and percentages. Ordinal parameters are presented as medians with interquartile range (IQR) and compared using the Wilcoxon rank-sum test. Continuous parameters are presented as means with standard deviation and compared by paired Student's t-test or Wilcoxon rank-sum test, as appropriate. Interrater agreement was assessed using Kendall’s τ for ordinal parameters and two-way mixed intraclass correlation coefficient (ICC) for continuous parameters. Correlation coefficients range from 0 to 1, with 0.01–0.2 indicating slight agreement, 0.21–0.4 fair, 0.41–0.6 moderate, 0.61–0.8 substantial, and 0.81–0.99 almost perfect agreement. Statistical analysis was performed with SPSS software (IBM SPSS Statistics for Windows, version 25.0, Armonk, NY, USA). A p-value < 0.05 was considered statistically significant.

## Data Availability

All data will be made available upon request in an anonymized manner (contact: Dr. Lukas Goertz, Lukas.goertz@uk-koeln.de).
